# Radioiodine-induced oxidative stress in patients with differentiated thyroid carcinoma and effect of supplementation with vitamins C and E and selenium (antioxidants)

**DOI:** 10.1590/2359-3997000000128

**Published:** 2016-02-11

**Authors:** Pedro Weslley Rosário, Kelly Cristina Siqueira Batista, Maria Regina Calsolari

**Affiliations:** 1 Santa Casa de Belo Horizonte Belo Horizonte MG Brasil Programa de Pós-Graduação, Santa Casa de Belo Horizonte, Belo Horizonte, MG, Brasil; 2 Santa Casa de Belo Horizonte Belo Horizonte MG Brasil Serviço de Endocrinologia, Santa Casa de Belo Horizonte, Belo Horizonte, MG, Brasil

**Keywords:** Thyroid cancer, radioiodine, oxidative stress, antioxidant

## Abstract

**Objective:**

: The objective of this study, in addition to confirming that therapy with ^131^I causes oxidative stress, was to evaluate the effect of supplementation with vitamins C and E and selenium on this phenomenon by measuring plasma 8-epi-PGF2a, a marker of lipid peroxidation.

**Subjects and methods:**

: Forty patients with thyroid cancer submitted to thyroidectomy, who received 3.7 GBq ^131^I after levothyroxine withdrawal, were selected; 20 patients did not receive (control group) and 20 patients received (intervention group) daily supplementation consisting of 2000 mg vitamin C, 1000 mg vitamin E and 400 µg selenium for 21 days before ^131^I. Plasma 8-epi-PGF2a was measured immediately before and 2 and 7 days after ^131^I.

**Results:**

: A significant increase in plasma 8-epi-PGF2a after ^131^I was observed in the two groups. The concentrations of 8-epi-PGF2α were significantly higher in the control group before and 2 and 7 days after ^131^I. The percentage of patients with elevated 8-epi-PGF2α was also significantly higher in the control group before and after ^131^I. Furthermore, the increase (percent) in 8-epi-PGF2α was significantly greater in the control group (average of 112.3% *versus* 56.3%). Only two patients (10%) reported side effects during supplementation.

**Conclusions:**

: Ablation with ^131^I causes oxidative stress which can be minimized by the use of antioxidants.

## INTRODUCTION

An increase in the incidence of differentiated thyroid carcinoma (DTC) has been observed over the last decades and many patients with these tumors receive radioiodine as part of their treatment (1-3). Exposure to radiation causes oxidative stress, a condition in which the production of free radicals exceeds the antioxidant capacity of the organism, which results in DNA damage and lipid and protein peroxidation. Some studies report an increase in the concentrations of malondialdehyde (MDA) (4-6) and 8-epi-PGF2a (7,8), markers of lipid peroxidation, in patients with DTC after ablation or therapy with ^131^I.

Oxidative stress is one of the mechanisms whereby ^131^I causes the destruction of normal thyroid cells and tumor cells (desired effect), but it is also responsible for side effects, including damage to the salivary glands (9). Attenuation of this process would therefore be interesting, but there are few clinical studies evaluating the effect of the use of antioxidants on the oxidative stress induced by ^131^I in patients with DTC (10).

The objective of this study, in addition to confirming that ablation with ^131^I in patients with DTC causes oxidative stress, was to evaluate the effect of supplementation with vitamins C and E and selenium on this process by measuring plasma 8-epi-PGF2a, a sensitive and specific marker of lipid peroxidation.

## SUBJECTS AND METHODS

### Design

This was a prospective study, with predefined (i) patient selection criteria, (ii) formation of groups, (iii) supplementation (micronutrients, doses, time of administration), and (iv) time of laboratory measurements and imaging methods. These criteria were rigorously followed throughout the study.

### Patients

Between May 2013 and March 2014, all patients with DTC aged 18 to 60 years, who were seen at our institution after total thyroidectomy and who would be submitted to ablation with ^131^I (first time), were recruited. Since the activity administered (7) and preparation for TSH elevation (8) can influence the intensity of oxidative stress induced by ^131^I, only patients receiving 100 mCi ^131^I after levothyroxine (L-T4) withdrawal were selected, thus ensuring the homogeneity of the sample. The indication, activity and preparation for ablation with ^131^I were defined by the attending physician. Patients presenting conditions that could alter 8-epi-PGF2a concentrations (malnutrition; obesity; diabetes; inflammatory, infectious or autoimmune diseases; kidney, liver or lung disease; heart failure; smoking; alcohol drinking; use of medications such as nonsteroidal anti- inflammatory drugs, corticosteroids, statins or vitamin supplements in the last 3 months) were excluded. The first 20 patients included did not receive supplementation (control group), while the following 20 patients received the supplements (intervention group). This study was approved by Ethical Committee of Santa Casa de Belo Horizonte, Brazil. All participants provided informed written consent.

### Supplementation

The patients received oral supplements consisting of daily doses of 2000 mg vitamin C, 1000 mg vitamin E and 400 µg selenium for 21 days before ^131^I. These micronutrients were chosen because they are potent antioxidants (11-13). The doses were based on respective safety limits (14,15) and the duration of supplementation was based on the time necessary to achieve maximum elevation in the serum levels of these micronutrients (16-19).

### Ablation with 131I

All patients discontinued L-T4 replacement therapy for 4 weeks and consumed a low-iodine diet for 10 days before ^131^I. Anterior and posterior whole-body images were obtained after 7 days (RxWBS). Eight months after ablation, the patients were submitted to neck ultrasonography (US) and measurement of thyroglobulin (Tg) and anti-Tg antibodies (TgAb) after stimulation of TSH elevation. An excellent response to initial therapy or complete ablation was defined when stimulated Tg < 1 ng/mL in the absence of TgAb and US showing no abnormalities (2,3).

### Evaluation of oxidative stress

Plasma 8-epi-PGF2a was measured immediately before and 2 and 7 days after ^131^I. These times were defined considering that oxidative stress occurs and continues at high intensity a few days after ^131^I (4-9).

## METHODS

Plasma 8-epi-PGF2a was measured with a specific enzyme immunoassay. Details of this assay have been published previously (8). The mean value plus 2 SDs was 22 pg/mL for a control group consisting of 24 (16 women and 8 men) healthy nonsmoking subjects aged 18-65 y (mean, 45 y) without known disease and not using any medications (8). Chemiluminescent assays were used for the measurement of Tg, TgAb, and TSH.

Doppler US was performed with a linear multifrequency transducer. US was defined as negative when it did not reveal suspicious lesions (20,21).

### Statistical analysis

Means were compared between groups by the Student t-test. The χ^2^ test was used to detect differences in the proportion of cases. ANOVA was used to compare 8-epi-PGF2a concentrations between the different time points. A P value of less than 0.05 was considered to be significant.

## RESULTS

### Patients

The groups (control and intervention) were similar in terms of sex, age, body mass index, tumor histology and stage, and TSH levels (Table 1).

**Table 1:** Characteristics of the patients studied

	Control group (n = 20)	Intervention group (n = 20)
Sex		
Female	16 (80%)	17 (85%)
Male	4 (20%)	3 (15%)
Age [range (mean), years]	21-60 (40.6)	21-60 (41)
BMI [range (mean), kg/m^2^]	19.2-28.9 (22.4)	19.3-29 (23.2)
Tumor		
Papillary	19 (95%)	18 (90%)
Follicular	1 (5%)	2 (10%)
TNM [2]		
T1bNx	2 (10%)	0
T1N1	1 (5%)	3 (15%)
T2Nx	6 (30%)	7 (35%)
T2N1	3 (15%)	3 (15%)
T3Nx	6 (30%)	3 (15%)
T3N1	2 (10%)	4 (20%)
TSH [range (mean), mIU/L]*	34-135 (79.4)	39-123 (78.5)
RxWBS		
Uptake in the thyroid bed	18 (90%)	19 (95%)
Ectopic cervical uptake	2 (10%)	1 (5%)
Uptake [range (mean), %]	0.5-1.8 (1.05)	0.6-1.7 (1.08)

*At the time of ^131^I administration.

BMI: body mass index; RxWBS: posttherapy whole body scanning.

### Plasma 8-epi-PGF2α (Figure 1)

**Figure 1:**
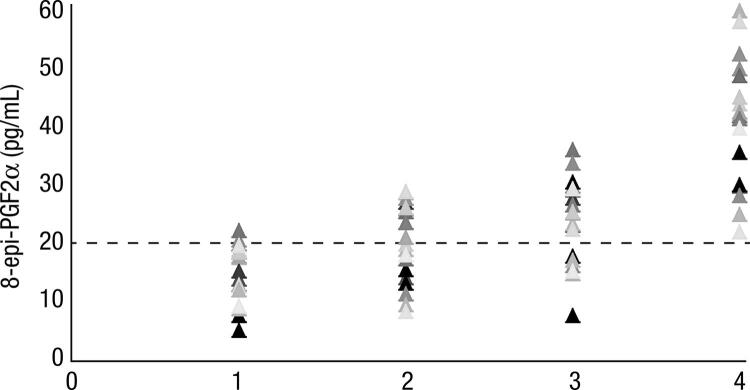
Plasma concentrations of 8-epi-PGF2α in the two groups: 1, intervention group (before 131I); 2, control group (before 131I); 3, intervention group (maximum value after 131I); 4, control group (maximum value after 131I). The dashed line indicates the upper limit of normal (22 pg/mL).

There was a significant increase in plasma 8-epi-PGF2α after ^131^I in the control (p < 0.0001) and intervention (p < 0.02) groups, without a difference in the results between 2 days *versus* 7 days after ^131^I (Table 2).

**Table 2:** Plasma concentrations of 8-epi-PGF2a in the two groups

	Control group (n = 20)	Intervention group (n = 20)	p-value
Before ^131^I [range (mean), pg/mL]	8.6-28.2 (20.5)	5.3-22.5 (15.7)	0.02
Elevated 8-epi-PGF2a (> 22 pg/mL)	9 (45%)	1 (5%)	0.01
2 days after ^131^I [range (mean), pg/mL]	19.8-60.3 (41.1)	7.8-36.6 (23.8)	0.001
7 days after ^131^I [range (mean), pg/mL]	22.3-62.2 (41.4)	7.1-34.1 (23.6)	0.001
Elevated 8-epi-PGF2a (> 22 pg/mL)^§^	20 (100%)	13 (65%)	0.05
Percent increase after ^131^I (mean)^§^	71%-159% (112.3%)	25%-130% (56.4%)	0.001

^§^ Considering the maximum value after^ 131^I.

When the two groups were compared, the concentrations of 8-epi-PGF2α were significantly higher in the control group before and 2 days and 7 days after ^131^I (Table 2). The percentage of patients with elevated 8-epi-PGF2α concentrations (> 22 pg/mL) was significantly higher in the control group before and after ^131^I (Table 2). Furthermore, the increase (percent) in 8-epi-PGF2α was significantly greater in the control group than in the intervention group (Table 2).

### Safety of supplementation

During supplementation, only two (10%) patients reported side effects; one patient had noninflammatory diarrhea in the last week and the other reported a metallic taste sensation one week after the beginning of supplementation, but did not require discontinuation.

### Response to ablation

In the two groups, 90% of the patients showed an excellent response to initial therapy or complete ablation.

## DISCUSSION

We first highlight that this was a prospective study. The selection criteria, including the same ^131^I activity and preparation for TSH elevation (L-T4 withdrawal), and patient assignment to each group permitted to obtain two highly similar groups. Additionally, the assessment protocol was uniform. These facts permit us to conclude that the differences in the results were due to the intervention performed (antioxidant supplementation). The choice of the marker is another important factor. 8-Epi-PGF2α is generated after free radical-mediated peroxidation of arachidonic acid, and some properties render this compound a reliable indicator of oxidative stress *in vivo* (22): it is a specific product of lipid peroxidation and a stable compound; it is present in detectable quantities in all normal biological fluids and tissues, allowing the definition of a normal range; its formation increases dramatically *in vivo* after several oxidant injuries; its formation is modulated by antioxidant status; its levels are not affected by dietary lipid content. 8-Epi-PGF2a is the best in vivo marker of lipid peroxidation, but few studies have evaluated oxidative stress associated with ablation or therapy with ^131^I in patients with DTC using this marker (7,8).

The results of the present study confirm that ablation with ^131^I causes oxidative stress (4-9). Only one study did not observe this fact (23). Although a direct association between the activity administered and the intensity of oxidative stress is expected, a significant increase in 8-epi-PGF2a has been observed even after a low ^131^I activity (7). It is also possible that the use of recombinant human TSH, avoiding hypothyroidism, causes less radioiodine-induced oxidative stress, but an increase in plasma 8-epi-PGF2a has been reported after ablation with ^131^I even when this preparation was used (8).

With respect to the use of antioxidants, to our knowledge, there are only two previous clinical studies. The first study compared MDA concentrations 4 days after radioiodine and 1 month after the daily use of 1000 mg vitamin C and showed a significant reduction (4); however, this reduction is also known to occur spontaneously (7), and it is not possible to ensure that the result was due to the administration of vitamin C. The second study demonstrated less functional impairment of the salivary glands for supplementation with 800 IU vitamin E/day 1 week before and 4 weeks after radioiodine, but markers of oxidative stress were not evaluated (10). The present results showed that combined supplementation with vitamins C and E and selenium was able to significantly attenuate the oxidative stress induced by radioiodine.

The present study has some limitations. It is not possible to draw conclusions regarding the contribution of each micronutrient to the result found, or regarding the possible additive or synergistic effect of their combined use. Adverse effects were observed in two patients of the intervention group, but we cannot rule out that they were due to the acute toxicity of ^131^I (2). Previous studies using vitamin C, vitamin E and selenium doses similar to those employed in the present study revealed no adverse clinical effects (24,25). Although the doses used were safe, we cannot rule out that the same effect could be achieved with lower doses.

Certainly the results of the present study will pave the way for clinical trials using micronutrients with antioxidant activity as strategies to minimize the side effects of ^131^I. Moreover, although oxidative stress is one of the factors responsible for the desired effects of radioiodine, apparently the modulation of this process did not compromise the efficacy of ablation.

## CONCLUSIONS

Ablation with ^131^I promotes oxidative stress which can be attenuated by supplementation with antioxidants. Further studies are needed to define whether this measure is able to reduce adverse clinical events.
